# Characteristics of established KSG cells derived from the scorpionfish *Sebastiscus marmoratus*: what happens under the hydrostatic pressure like the deep sea?

**DOI:** 10.1007/s11626-013-9723-0

**Published:** 2014-01-08

**Authors:** Yusuke Tsuruwaka, Eriko Shimada, Makiko Kikuchi, Yuji Hatada

**Affiliations:** 1 Marine Bioresource Exploration Research Team (MBE), Marine Biodiversity Research Program, Institute of Biogeosciences, Japan Agency for Marine-Earth Science and Technology (JAMSTEC), Yokosuka, 237-0061 Japan; 2 Division of Applied Biosciences, Graduate School of Agriculture, Kyoto University, Kyoto, 606-8502 Japan; 3 Department of Applied Material and Life Science, Kanto Gakuin University, Yokohama, 236-8501 Japan

**Keywords:** KSG cell, Serum-free culture, Hydrostatic pressure, SeaGrow, Hy-Fish

## Abstract

Advances in cell biology depend, partly, on the development of new cell lines and culture methods. Our research focused on a fibroblast-like cell line, “KSG,” which is derived from scorpionfish fin tissue (*Sebastiscus marmoratus*). Cells were grown in Leibovitz’s L-15 medium with 10% fetal bovine serum following standard procedures. The optimum growth temperatures for these lines ranged from 15°C to 25°C. All cells survived storage for at least 3 yr at −80°C. Subsequently, they were continuously cultured until the 78th generation without evident changes in their morphology. Moreover, we were able to culture KSG cells in the absence of fetal bovine serum in a culture medium containing the fish serum “SeaGrow.” Optimum SeaGrow concentrations for these cells ranged from 5% to 20%. The growth rate of KSG cells decreased when the concentration of SeaGrow was reduced to 1%. However, this decrease could be partially reversed by adding 0.5% “Hy-Fish.” In addition, the inclusion of Hy-Fish improved cell adhesion. KSG cells that were cultured in serum-free culture media containing 0.5% and 1% Hy-Fish had been added and were able to survive at low densities. Furthermore, we successfully transfected this cell line with a commercial plasmid vector coding a fluorescent protein using the cationic lipid. Finally, the analyses of cell behavior under hydrostatic pressure showed that some pressures (10 MPa) helped the cells to proliferate more.

**Electronic supplementary material:**

The online version of this article (doi:10.1007/s11626-013-9723-0) contains supplementary material, which is available to authorized users.

## Introduction

Analyses of cell proliferation using cultured cells provide useful information for determining the functions of tissues and organs as well as defining the process of cell differentiation and embryo development (Ciemerych et al. 2011). Abnormal cell growth can result in the development of cancerous cells or programmed cell death (i.e., apoptosis). Cultured cell lines have been widely used to investigate the factors controlling these processes (Wolf and Quimby 1969). Cultured cells are typically incubated in blood serum containing multiple growth factors or a synthetic medium with purified growth factors. In mammalian cell culture, a range of factors that regulate cell proliferation have been identified and their functional mechanisms documented (Barnes and Sato 1980). In fish, researchers have used cultured cell lines to study fish viruses (Yan et al. 2011). Cells are cultivated from embryos, gonads, kidneys, and fins and are typically bathed in a commercially available synthetic medium containing 10–20% fetal bovine serum (FBS). Fish serum is thought to promote higher growth in teleost cells than FBS; however, the growth factors associated with this response have not been identified (Hashimoto et al. 1997). Although the cells of ectotherms, including fish, are expected to grow in the temperature ranges suitable for adult individuals, the regulatory mechanisms remain unknown (Hightower and Renfro 1988; Bols et al. 1992; Buonocore et al. 2006). At present, all established fish cell lines, including the KSG scorpionfish cell line, are cultured in media containing FBS. Since the outbreak of bovine spongiform encephalopathy at the beginning of 2000, there has been an emphasis on developing a culture method that does not use mammalian-derived components (Padilla et al. 2011). We investigated the usefulness of cell culture using serum derived from fish (Salmonidae) and evaluated the effectiveness of fish-derived culture medium additives. Furthermore, we examined the unique fish cell line characteristics: an essential relation between the hydrostatic pressure and the cell proliferation.

## Materials and Methods

### Preparation of the cell line.

The scorpionfish is distributed throughout the marine areas around Japan at depths of ≈5–200 m. We captured scorpionfish near Fukuura Wharf in Yokohama City, Kanagawa Prefecture, Japan and prepared the KSG cells as described previously (Japan Patent Kokai 2008). In brief, we cultured the caudal fin tissue in Leibovitz’s L-15 (MP Biomedicals, Santa Ana, CA) culture medium, with 10% FBS (MP Biomedicals), 4 g/L NaCl (Wako, Osaka, Japan), and 1% penicillin-streptomycin (MP Biomedicals). The caudal fin tissue was cut into 1-mm squares, immersed in the medium, and cultured in an incubator at 25°C. After 3 d, we confirmed the migration of the fibroblasts. Even after the tissue was removed, the fibroblasts continued to multiply. We have confirmed survival of the fibroblasts to the 78th generation. We observed cell morphology using an inverted microscope CKX41 (Olympus, Tokyo, Japan) connected to a digital camera ARTCAM-130MI (Armssystem, Tokyo, Japan).

### Culture method using FBS.

The scorpionfish KSG cell line was seeded into 25-cm^2^ tissue culture flasks and cultured in Leibovitz’s L-15 medium containing 10% FBS at 25°C. Cells that reached the confluent state (500 cells/mm^2^) were separated with TrypLE Express (Gibco, Carlsbad, CA), seeded into new 25-cm^2^ tissue culture flasks, and subcultured. We replaced the culture medium with new medium every 3 d.

### Culture method using fish serum.

KSG cells in frozen storage were thawed and cultured in Leibovitz’s L-15 medium containing 10% FBS. Then, the culture medium was substituted for a medium containing Salmonidae fish serum, SeaGrow (EastCoast Biologics, North Berwick, ME). We adjusted the concentration of heat-inactivated SeaGrow to 5%, 10%, and 20%. The KSG cell line cultured with SeaGrow is hereafter referred to as KSG-SeaGrow. We evaluated the effect of the concentration of SeaGrow on growth rate (doubling time) and cell adhesion in the KSG-SeaGrow cell line. The cells were counted using an automated cell counter TC10 (Bio-Rad, Hercules, CA). The time taken for the number of cells to double (doubling time, DT) was calculated using the following formula:

$$ \mathrm{Doubling}\;\mathrm{time}\;\left(\mathrm{DT}\right)=\left(T-{T}_0\right) \log 2/\left( \log N- \log {N}_0\right) $$

*T*: time [h]
*N*: cell density [cells/mm^2^]
*T*_0_: initial time [h]
*N*_0_: initial density [cells/mm^2^]

### Effect of a fish-derived culture medium additive on growth rate.

Hy-Fish (Maruhachi Muramatsu, Shizuoka, Japan) is a component extracted from the bony parts of skipjack tuna. The extract was developed as a culture medium additive for culturing mammalian cells. We investigated whether KSG-SeaGrow cells could be cultured in media containing low concentrations of SeaGrow and Hy-Fish. We added 0.5% Hy-Fish to culture media containing SeaGrow (1% or 2.5%) and measured growth rate and cell adhesion in the KSG-SeaGrow cell line as mentioned above.

### Serum-free culture using fish-derived culture medium additive.

We cultured KSG-SeaGrow cells using Leibovitz’s L-15 culture medium without serum and containing 0.1%, 0.5%, or 1.5% Hy-Fish. We measured growth rate and monitored cell adhesion as stated above.

### Transfection of KSG cells.

KSG-SeaGrow cells were transfected with the pCDNA3.1/Zeo plasmid (Invitrogen, Carlsbad, CA), which contains a CMV promoter, a SV40 polyadenylation signal, a gene conveying resistance to zeocin, and a multicloning site inserting a yellow cameleon 2.12 (YC2.12) sequence. This plasmid is hereafter referred to as pCDNA3.1-YC2.12. Constructs of YC2.12 were kindly provided by A. Miyawaki (RIKEN, Wako, Japan). Following transfection, YC2.12 is expressed as a cyan and a yellow variant (YFP) of the green fluorescent protein. We transfected pCDNA3.1-YC2.12 into KSG-SeaGrow cells, which had attained a 70% confluent state using lipofectamine2000 (Invitrogen), following the manufacturer’s instructions. The cells were then incubated for 24 h at 25°C. Positive cells were selected by continuous incubation in Leibovitz’s L-15 medium containing zeocin (Invitrogen) at 0, 50, 100, 200, 400, 600, 800, or 1,000 μg/ml, added 48 h after transfection. The culture medium containing zeocin was changed every 3 d, and the experiment was conducted over a period of ≈1 mo. We observed YFP expression in the positive cells using a confocal fluorescence microscope FV5-PSU + IX71 (Olympus) connected to a digital camera ORCA-ER (Hamamatsu Photonics, Shizuoka, Japan). Methods for quantifying fluorescence have been described previously (Tsuruwaka et al. 2007). However, during this experiment, we focused solely on YFP detection.

### KSG cell proliferation under hydrostatic pressure.

KSG cells attached to the collagen-coated, pressure-resistant glass were placed in a high-hydrostatic pressure chamber (ABLE Corporation, Tokyo, Japan). A detailed chamber system was reported by Koyama et al. (2001). Amount of pressure is indicated with megapascals (MPa) here. Atmospheric pressure is nearly equal to 0.1 MPa = 0.9869 atm = 1.0197 kg of force/cm^2^. When water depth gets deeper by 10 m, hydrostatic pressure rises by 0.1 MPa. The cell morphological feature and proliferation were examined under pressures from 0 to 40 MPa after 20 min with the differential interference microscope IX70 (Olympus) connected to a CCD camera M-3204C (Olympus). Twenty minutes later, the pressure was released to atmospheric pressure. Then, the cell was cultured at 25°C, and the proliferation rate was measured as stated above.

## Results and Discussion

### Culture method using fish serum.

**Figure 1.**
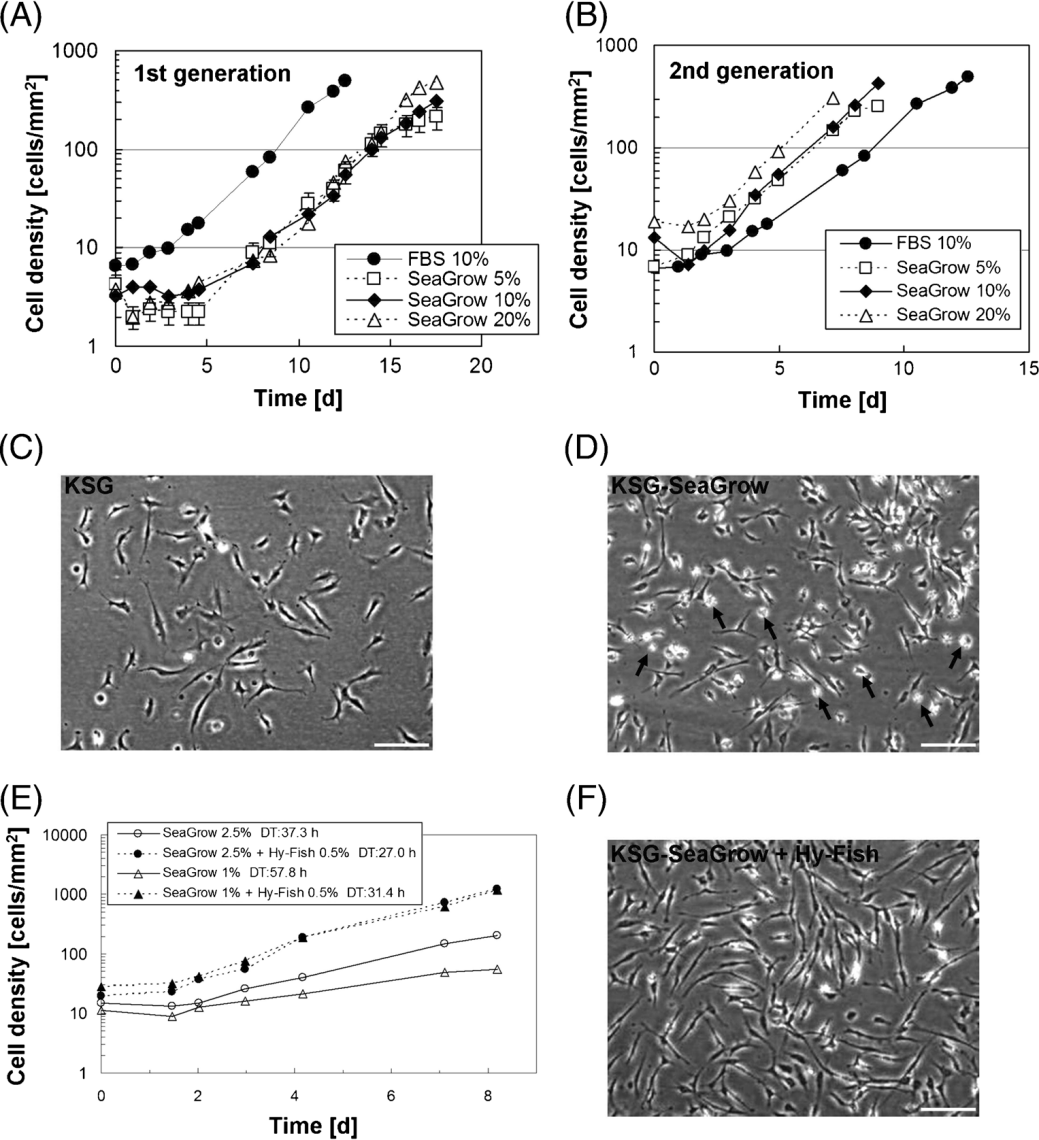
The KSG cell growth rate and morphological features. (*A*) The growth curves of first-generation KSG cultured in FBS and of KSG-SeaGrow cultured in a range of concentrations of SeaGrow. (*B*) The growth curve of the second-generation KSG/KSG-SeaGrow after subculturing. (*C*) Cell shapes of KSG. *Scale bar*, 50 μm. (*D*) Cell shapes of KSG-SeaGrow. A few cells were peeled (*arrow*). *Scale bar*, 50 μm. (*E*) The growth curve of KSG-SeaGrow cultured by adding Hy-Fish to SeaGrow serum culture media. (*F*) Cell shapes of KSG-SeaGrow + Hy-Fish. *Scale bar*, 50 μm.

Because of the effects of changing serum, the first-generation KSG-SeaGrow cells passed through a lag phase of approximately 100 h before entering the multiplication phase (Fig. 1*A*). The doubling time was equal to that of KSG cells (≈40 h). The second and subsequent generations of KSG-SeaGrow began multiplying immediately after adhesion, and the doubling time was shorter than KSG and was stable at 30 h (Fig. 1*B*). We observed many instances where the area around the KSG-SeaGrow cells appeared white in color, or where the cells were round due to uplifting, and we found that KSG-SeaGrow cells had more of these uplifted cells compared with KSG cells. These uplifted cells were not sufficiently extended, resulting in weak adhesion of the KSG-SeaGrow line (Fig. 1*C*, *D*). We were unable to determine whether the uplifting was due to the inclusion of SeaGrow or whether the cells required more time to adjust to the substitution of SeaGrow for FBS. Since the second and subsequent generations of KSG-SeaGrow multiplied faster than those of KSG (Fig. 1*B*), cells that have undergone more subcultivations in KSG-SeaGrow may ultimately adapt to continuous growth in SeaGrow.

### Effect of a fish-derived culture medium additive on growth rate.

The cell doubling time was 57.8 h when KSG-SeaGrow was cultured with 1% SeaGrow, but decreased to 31.4 h with the addition of 0.5% Hy-Fish (Fig. 1*E*). Figure 1*F* shows a photograph of cells cultured in 0.5% Hy-Fish and 2.5% SeaGrow. Cell adhesion was improved by adding Hy-Fish to the SeaGrow serum culture medium. We successfully cultured fish cells using SeaGrow and/or Hy-Fish in the place of mammalian-derived constituents such as FBS.

### Serum-free culture using a fish-derived culture medium additive.

**Figure 2.**
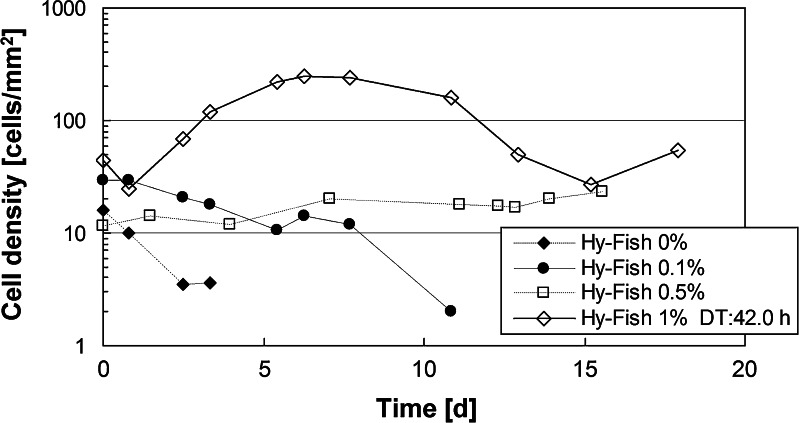
The growth curve of KSG-SeaGrow in serum-free culture medium with Hy-Fish added.

When KSG-SeaGrow cells were cultured in serum-free Leibovitz’s L-15 culture medium to which Hy-Fish had not been added, there was no multiplication after adhesion was confirmed. In addition, we observed that all cells detached on the fourth day after seeding. The addition of 0.1% Hy-Fish delayed detachment of all cells until the 12th day. At a concentration of 0.5% Hy-Fish, the cells survived for 19 d while remaining at a low density (20 cells/mm^2^) (Fig. 2). At 1% Hy-Fish, the cell density increased to ≥200 cells/mm^2^ and then decreased to 20 cells/mm^2^. Interestingly, we did not observe cell adhesion at a concentration of 5% Hy-Fish. According to the exposure experiment in Konishi et al. (2006), low concentration of stimulants such as alcohols and heavy metals demonstrated higher activation temporarily in fish. A similar mechanism may have been involved in the cell growth with the presence of 1% Hy-Fish in the KSG cells. Taken together, our results suggest that it is feasible to culture KSG cells in a serum-free environment by supplementing the media with Hy-Fish.

### Yellow fluorescent protein expression in KSG-SeaGrow.

**Figure 3.**
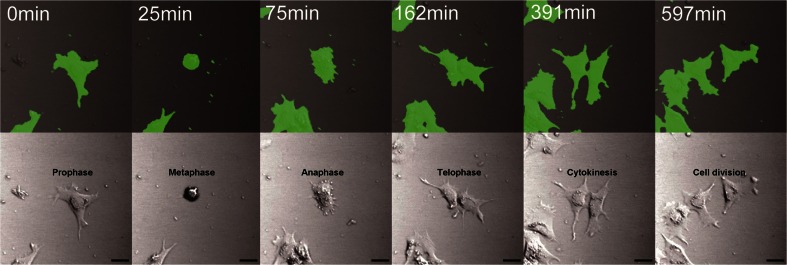
Expression of the YC2.12 gene in KSG-SeaGrow during division. The transfected cells divided normally (*upper*, fluorescence images; *lower*, bright field images). *Scale bars*, 20 μm.

The next question we need to consider is what promoter would function in KSG-SeaGrow. Whether the CMV promoter can be worked was then investigated. As a result, KSG-SeaGrow was successfully transfected with the pCDNA3.1-YC2.12 plasmid using lipofectamine2000. We detected YFP expression 48 h after transfection. Fluorescence of yellow cameleon was observed in ≈40% of KSG-SeaGrow cells at this stage. Following chemical resistance selection using zeocin, the cells were all fluorescent and normally divided (Fig. 3). We obtained similar results using KSG cells, which were cultured with FBS (data not shown). The expression of pCDNA3.1-YC2.12 by KSG/KSG-SeaGrow cells suggests that it is possible to use a widely available, commercial CMV promoter.

### KSG cell behavior under the hydrostatic pressure.

**Figure 4.**
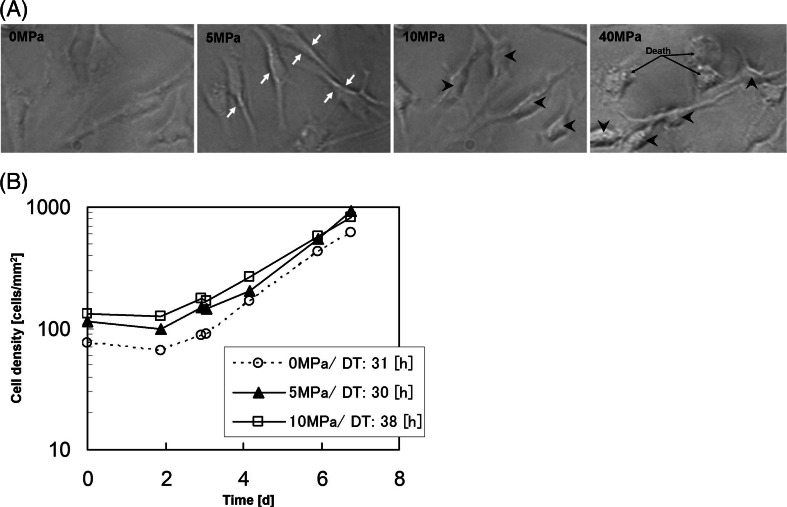
KSG cell morphology under the hydrostatic pressure. (*A*) KSG cells given 0–40 MPa. *Arrows*: protruded cells; *arrow heads*: round cells. (*B*) The growth curve of KSG under 0–10 MPa.

Fish are always stimulated by the hydrostatic pressures. Here, we examined the behavior of cell proliferation under hydrostatic pressure. Figure 4*A* shows that the KSG cells under 5 MPa (equivalent to 500-m depth) showed cell protrusions. Then, less protrusions and a round shape were observed in the cells under 10 MPa (equivalent to a 1,000-m depth). In those under 20–40 MPa, however, each cell aggregated and became round in shape (Supplemental Fig. 1). Also, some of the cells detached from the culture plate. Moreover, the cells subjected to various pressures were cultured to investigate the cell density. The cells given 5 and 10 MPa showed higher cell density than 0 MPa (Fig. 4*B*). Those under ≥20 MPa did not attach on the culture flasks. These results suggest that 10-MPa hydrostatic pressures help the cells to proliferate more.

Results in this study show that KSG scorpionfish cells give a choice of Leibovitz’s L-15 culture media additive: mammalian-derived serum (FBS), fish serum (SeaGrow), or fish bone extraction (Hy-Fish). The KSG cell line is an interesting research platform for gene and protein expression studies to elucidate deep-sea fishes.

## Electronic supplementary material

Below is the link to the electronic supplementary material.

Supplemental Fig. 1 (MOV 6478 kb)
